# Introduction to phasing

**DOI:** 10.1107/S0907444910006694

**Published:** 2010-03-24

**Authors:** Garry L. Taylor

**Affiliations:** a Centre for Biomolecular Sciences, University of St Andrews, St Andrews, Fife KY16 9ST, Scotland

**Keywords:** phasing

## Abstract

When collecting X-ray diffraction data from a crystal, we measure the intensities of the diffracted waves scattered from a series of planes that we can imagine slicing through the crystal in all directions. From these intensities we derive the amplitudes of the scattered waves, but in the experiment we lose the phase information; that is, how we offset these waves when we add them together to reconstruct an image of our molecule. This is generally known as the ‘phase problem’. We can only derive the phases from some knowledge of the molecular structure. In small-molecule crystallography, some basic assumptions about atomicity give rise to relationships between the amplitudes from which phase information can be extracted. In protein crystallography, these *ab initio* methods can only be used in the rare cases in which there are data to at least 1.2 Å resolution. For the majority of cases in protein crystallography phases are derived either by using the atomic coordinates of a structurally similar protein (molecular replacement) or by finding the positions of heavy atoms that are intrinsic to the protein or that have been added (methods such as MIR, MIRAS, SIR, SIRAS, MAD, SAD or com­binations of these). The pioneering work of Perutz, Kendrew, Blow, Crick and others developed the methods of isomorphous replacement: adding electron-dense atoms to the protein without disturbing the protein structure. Nowadays, methods from small-molecule crystallography can be used to find the heavy-atom substructure and the phases for the whole protein can be bootstrapped from this prior knowledge. More recently, improved X-ray sources, detectors and software have led to the routine use of anomalous scattering to obtain phase information from either incorporated selenium or intrinsic sulfurs. In the best cases, only a single set of X-ray data (SAD) is required to provide the positions of the anomalous scatters, which together with density-modification procedures can reveal the structure of the complete protein.

## Abstract 2

This introductory paper to the CCP4 weekend on experimental phasing introduces the concept of the ‘phase problem’ for non-experts. Modern methods of phasing are explored, including some recent examples that can be downloaded as tutorials.

## 1. Introduction

### 1.1. Phasing

There are many excellent comprehensive texts on macromolecular crystallography that include sections on phasing methods (Blundell & Johnson, 1976 ▶; Drenth, 1994 ▶, 2006 ▶; Blow, 2002 ▶; Lattman & Loll, 2008 ▶; Rhodes, 2006 ▶; McPherson, 2009 ▶; Rossmann & Arnold, 2001 ▶; Rupp, 2009 ▶). This introduction to the CCP4 Study Weekend on Experimental Phasing attempts to give an overview of phasing for those new to the field. Many entering protein crystallography come from a biological background and are unfamiliar with the details of Fourier summation and complex numbers. The routine incorporation of selenomethionine into proteins, the wide availability of synchrotrons and improvements in detector technology and in software mean that in many cases structure solution has become ‘black box’. Not all structure solutions are plain sailing, however, and it is still useful to have some understanding of phasing. Here, we will emphasize the importance of phases, describe how phases are derived from some prior knowledge of structure and look briefly at phasing methods (direct, molecular replacement and heavy-atom isomorphous replacement). In most heavy-atom phasing methods the aim is to preserve isomorphism, such that the only structural change upon heavy-atom substitution is local and there are no changes in unit-cell dimensions or the orientation of the protein in the cell. Single-wavelength and multiwavelength anomalous diffraction (SAD/MAD) experiments normally achieve this as in the absence of radiation damage iso­morphism is preserved when all diffraction data are collected from a single crystal. Where non-isomorphism does occur, this can be used to provide phase information and we will look at an example in which non-isomorphism was used to extend phases from 6 to 2 Å.

**Figure 1:**
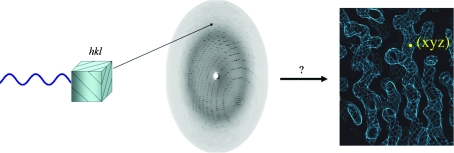
The diffraction experiment.

In the diffraction experiment (Fig. 1 ▶), we measure on a detector the intensities of waves scattered from planes (denoted by *hkl*) in the crystal. The intensity value is a measure of the number of electrons present in one particular plane. The amplitude of the wave |*F*_*hkl*_| is proportional to the square root of the intensity. To calculate the electron density at a position (*xyz*) in the unit cell of a crystal we need to perform the following summation over all the *hkl* planes. In words, we can express this as the electron density at (*xyz*) is the sum of the contributions to the point (*xyz*) of a wave scattered from a plane (*hkl*) whose amplitude depends on the number of electrons in the plane added with the correct relative phase relationship or, mathematically, 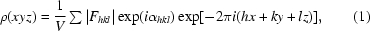
where *V* is the volume of the unit cell and α_*hkl*_ is the phase associated with the structure-factor amplitude |*F*_*hkl*_|. We can measure the amplitudes, but the phases are lost in the experiment. This is the phase problem.

### 1.2. The importance of phases

**Figure 2:**
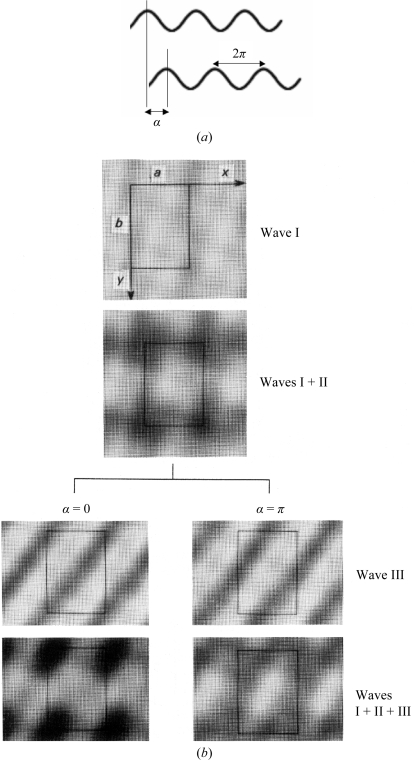
(*a*) The definition of a phase angle α. (*b*) The result of adding three waves, where the third wave is added with two different phase angles.

**Figure 3:**
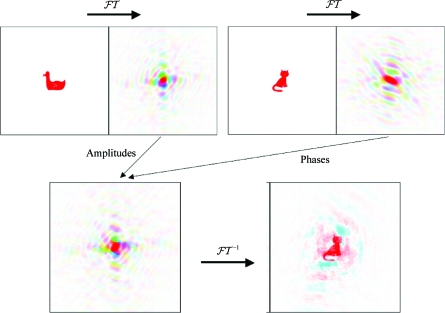
The importance of phases in carrying information. Top, the diffraction pattern, or Fourier transform (FT), of a duck and of a cat. Bottom left, a diffraction pattern derived by combining the amplitudes from the duck diffraction pattern with the phases from the cat diffraction pattern. Bottom right, the image that would give rise to this hybrid diffraction pattern. In the diffraction pattern, different colours show different phases and the brightness of the colour indicates the amplitude. Reproduced courtesy of Kevin Cowtan.

The importance of phases in pro­ducing the correct electron density, or structure, is illustrated in Figs. 2 ▶ and 3 ▶. In Fig. 2 ▶ three ‘electron-density waves’ are added in a unit cell, which shows the dramatically different electron density resulting from adding the third wave with a different phase angle. In Fig. 3 ▶, from Kevin Cowtan’s *Book of Fourier* (http://www.ysbl.york.ac.uk/~cowtan/fourier/fourier.html), the importance of phases in carrying structural information is beautifully illustrated. The cal­culation of an ‘electron-density map’ using amplitudes derived from the diffraction of a duck and phases derived from the diffraction of a cat results in a cat: the phases carry much more information.

## 2. Recovering the phases

There is no formal relationship between the amplitudes and their phases; the only relationship is *via* the molecular structure or electron density. Therefore, if we can assume some prior knowledge of the electron density, or structure, this can lead to values for the phases. This is the basis for all phasing methods, including phase improvement or density modification (Table 1 ▶).

### 2.1. Direct methods

Direct methods are based on the positivity and atomicity of electron density that leads to phase relationships between the (normalized) structure factors, for which Hauptmann and Karle shared the 1985 Nobel Prize in Chemistry (see their Nobel lectures at http://nobelprize.org/nobel_prizes/chemistry/laureates/1985/). The triplet relation (2) shows how the phases of three reflections are related. For example, consider the case where **h** is the (2, 3, 5) reflection and **h**′ is the (1, 0, 3) reflection, such that **h** − **h**′ is therefore (1, 3, 2). The triplet relationship shows that the sum of the phases of the (−2, −3, −5), (1, 0, 3) and (1, 3, 2) reflections is approximately zero. Therefore, knowing the phases of two reflections allows one to derive the phase of a third. The tangent formula (3) is an equation derived for phase refinement based on the triplet relationship,
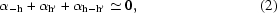

               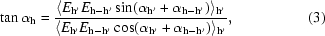
where *E* represents the normalized structure-factor amplitude; that is, the amplitude that would arise from point atoms at rest. Such equations imply that once the phases of some reflections are known, or can be given a variety of starting values, then the phases of other reflections can be deduced, leading to a bootstrapping to obtain phase values for all reflections. The requirement of what is for proteins very high-resolution data (<1.2 Å) has limited the usefulness of *ab initio* phase determination in protein crystallography, although direct methods have been used to phase small proteins (up to ∼1000 atoms). This high-resolution requirement of 1.2 Å, or the so-called Sheldrick’s rule (Sheldrick, 1990 ▶), has been given a structural basis with respect to proteins (Morris & Bricogne, 2003 ▶). However, direct methods are routinely used to find the heavy-atom substructure by programs such as *Shake-and-Bake* (*SnB*; Miller *et al.*, 1994 ▶), *SHELXD* (Sheldrick, 2008 ▶), *ACORN* (Foadi *et al.*, 2000 ▶) and *HySS* (Grosse-Kunstleve & Adams, 2003 ▶).

### 2.2. Molecular replacement (MR)

**Figure 4:**
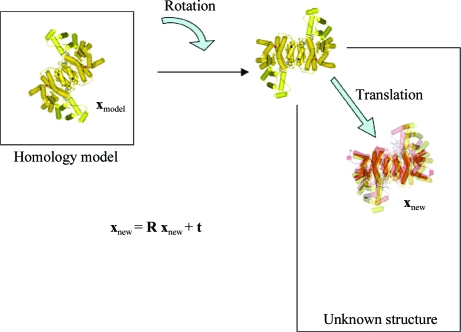
The process of molecular replacement.

When a structurally similar model is available, molecular replacement can be successful, using methods first described by Michael Rossmann and David Blow (Rossmann & Blow, 1962 ▶). As a rule of thumb, a sequence identity of >25% is normally required together with an r.m.s. deviation of <2.0 Å between the C^α^ atoms of the model and the new structure, although there are exceptions to this. Molecular replacement usually employs the Patterson function. A Patterson map is calculated using the same Fourier summation that is used to calculate an electron-density map but with (*F*_*hkl*_)^2^, or intensities, as the coefficients and therefore does not require knowledge of the phases. The resulting map is the convolution of the electron density with itself and provides a map that has peaks at interatomic vectors rather than at absolute atomic positions. A Patterson map can also be calculated using amplitudes calculated from the atomic coordinates of a structurally similar model and rotated over a Patterson map calculated from the structure-factor amplitudes of the new crystal to obtain the orientation of the model in the new unit cell. The translation of the correctly oriented model relative to the origin of the new unit cell can be found using similar Patterson methods through a search for vectors between symmetry-related molecules in the new unit cell, although other methods can be employed (Fig. 4 ▶).

### 2.3. Isomorphous replacement

**Figure 5:**
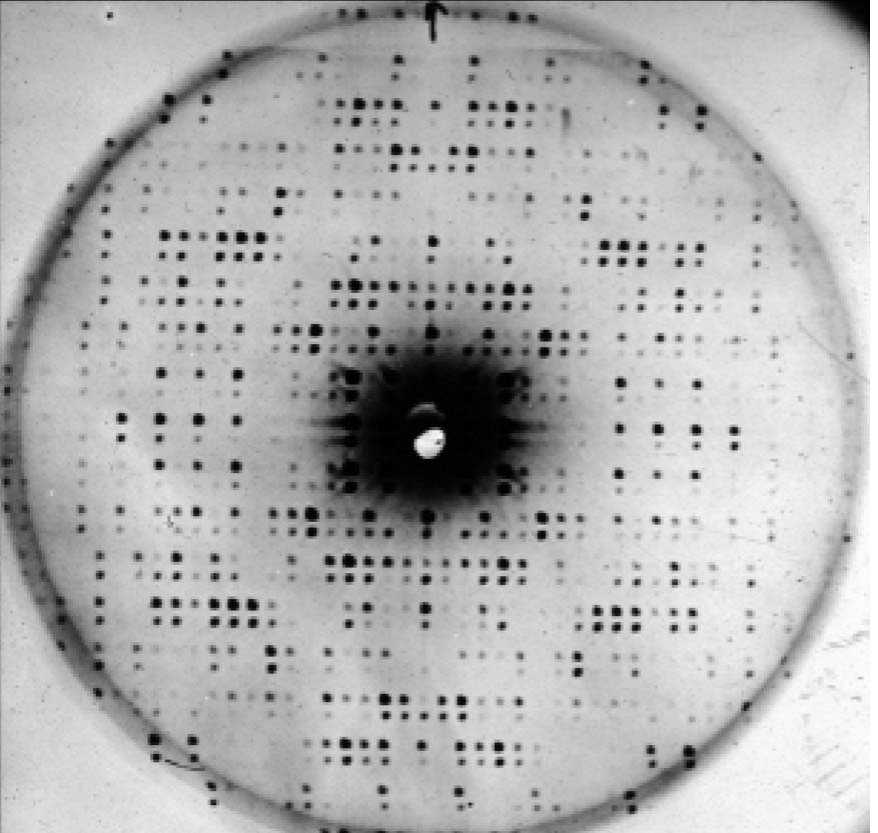
Two protein diffraction patterns superimposed and shifted vertically relative to one another. One is from native bovine β-lactoglobulin and the other is from a crystal soaked in a mercury-salt solution. Note the intensity changes for certain reflections and the identical unit cells (spacing of the spots) suggesting isomorphism. (Photograph courtesy of Professor Lindsay Sawyer.)

The use of heavy-atom substitution to solve the phase problem was invented very early on by small-molecule crystallographers, for example the isomorphous crystals (same unit cells) of CuSO_4_ and CuSeO_4_ (Groth, 1908 ▶). The changes in intensities of some classes of reflections were used by Beevers & Lipson (1934 ▶) to locate the Cu and S atoms. It was Max Perutz and John Kendrew who first applied the method to proteins (Perutz, 1956 ▶; Kendrew *et al.*, 1958 ▶) by soaking protein crystals in heavy-atom solutions to create isomorphous heavy-atom derivatives (same unit cell, same orientation of the protein in cell), which gave rise to measurable intensity changes that could be used to deduce the positions of the heavy atoms (Fig. 5 ▶).

Francis Crick is best known for his contribution to the structure of DNA, but he also made several contributions to macromolecular crystallography, in­cluding estimating the magnitude of the expected changes in the intensities of the reflections in isomorphous replacement (Crick & Magdoff, 1956 ▶). For example, the addition of a single Hg atom to a protein of 1000 atoms is predicted to produce an average fractional change of intensity of 25% using the formula
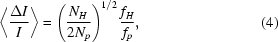
where *N*_*H*_ and *f*_*H*_ are the number of heavy atoms and their scattering factor at sinθ = 0° and *N*_*p*_ and *f*_*p*_ are the number of light atoms and their scattering factor at sinθ = 0°, respectively. The same paper also shows that for a 100 Å cubic unit cell a 0.5% change in unit-cell dimensions or a 0.5° rotation of the molecule within the unit cell would produce an average 15% change in intensity. Isomorphism is therefore critical.

**Figure 6:**
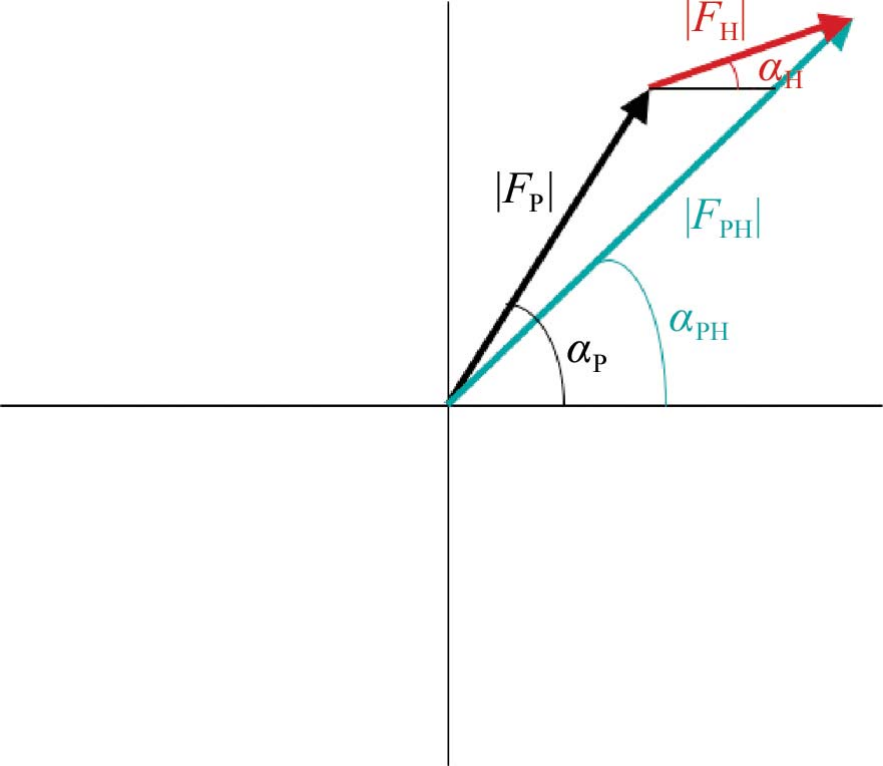
Argand diagram for SIR. |*F*_P_| is the amplitude of a reflection for the native crystal and |*F*_PH_| is that for the derivative crystal.

**Figure 7:**
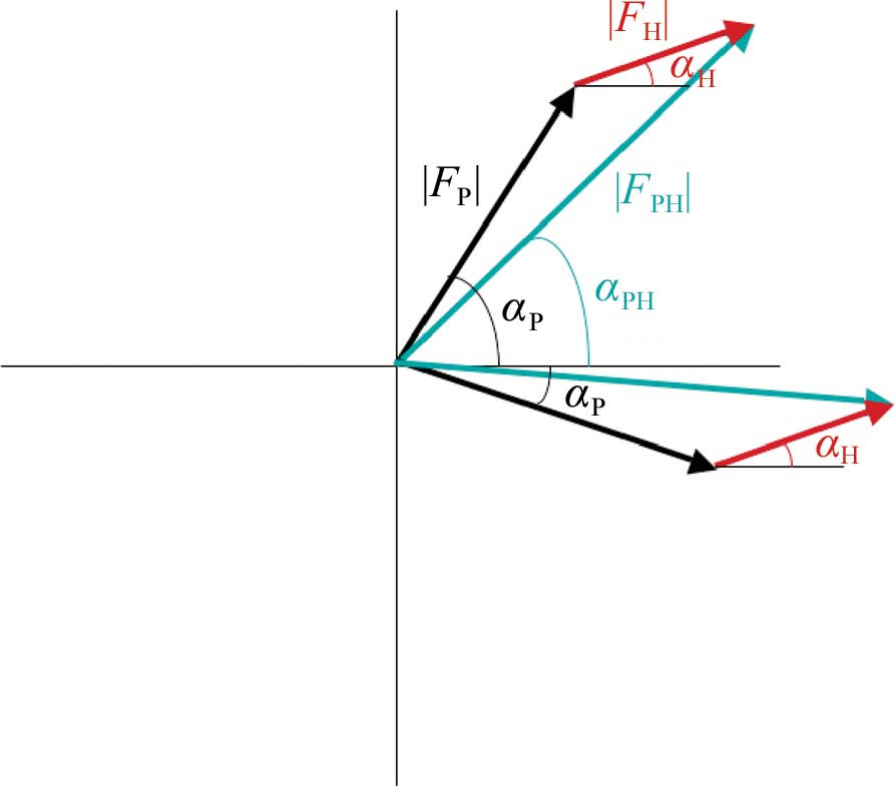
Estimation of the native protein phase for SIR.

In the case of a single isomorphous replacement (SIR) experiment, the contribution of the added heavy atom to the structure-factor amplitude and phases is best illustrated on an Argand diagram, which shows a plot of the real and imaginary axes of the complex plane (Fig. 6 ▶). The amplitudes of a reflection are measured for the native crystal, |*F*_P_|, and for the derivative crystal, |*F*_PH_|. The isomorphous difference, |*F*_H_| ≃ |*F*_PH_| − |*F*_P_|, can be used as an estimate of the heavy-atom structure-factor amplitude to determine the heavy atom’s positions using Patterson or direct methods. Once located, the heavy-atom parameters (*xyz* positions, occupancies and Debye–Waller thermal factors *B*) can be refined and used to calculate a more accurate |*F*_H_| and its corresponding phase α_H_. The native protein phase, α_P_, can be estimated using the cosine rule (Fig. 7 ▶),
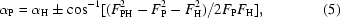
leading to two possible solutions symmetrically distributed about the heavy-atom phase.

**Figure 8:**
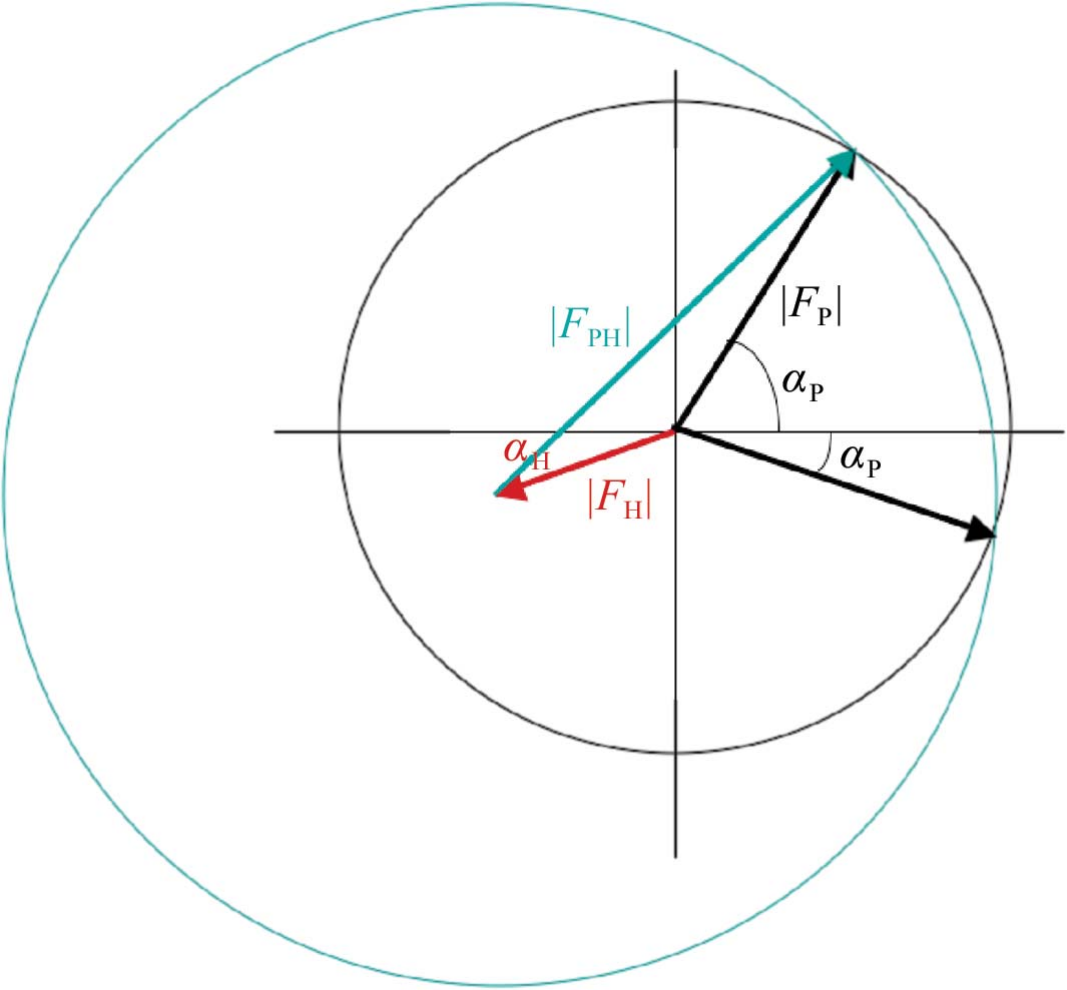
Harker construction for SIR.

This phase ambiguity is better illustrated in the Harker construction (Fig. 8 ▶). The two possible phase values occur where the circles intersect. The problem then arises as to which phase to choose. This requires a consideration of phase probabilities.

## 3. Phase probability

**Figure 9:**
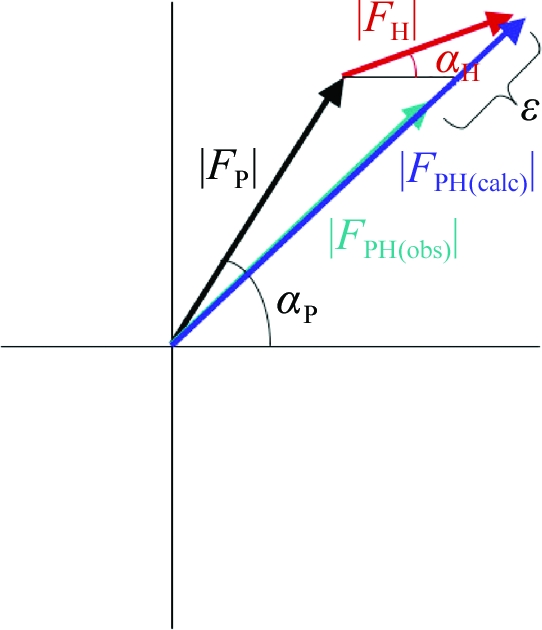
The lack of closure.

**Figure 10:**
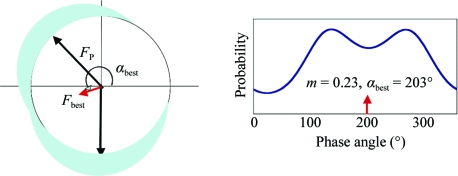
Phase probability for one reflection in an SIR experiment. *F*_best_ is the centroid of the distribution. The map calculated with |*F*_best_|exp(*i*α_best_) [or *m*|*F*_P_|exp(*i*α_best_)〈cosΔα〉, where *m* is the figure of merit] has least error. *m* = 0.23 implies a 76° phase error, since cos(76) = 0.23.

In reality, there are errors associated with the measurements of the structure factors, scaling and non-isomorphism errors, and errors in the derived heavy-atom positions and their occupancies, such that the vector triangle of Fig. 6 ▶ seldom closes. David Blow and Francis Crick (Blow & Crick, 1959 ▶) introduced the concept of lack of closure ∊ (6) and its use in defining a phase probability (7) (Fig. 9 ▶),
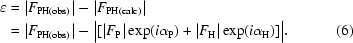
Making the assumption that all the errors reside in *F*_PH(calc)_ and that errors follow a Gaussian distribution, the probability of a phase having a certain value is then
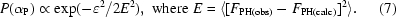
One could, for example, calculate such a probability from 0° to 360° in 10° intervals to produce a phase-probability distribution, the shape of which can be represented by four coefficients of a polynominal: the so-called Hendrickson–Lattman coefficients HLA, HLB, HLC and HLD (Hendrickson & Lattman, 1970 ▶). Blow and Crick also showed that an electron-density map calculated with a weighted amplitude representing the centroid of the phase distribution gave the least error. Fig. 10 ▶ shows the phase probability distribution for one reflection from an SIR experiment. The centroid of the distribution is denoted by *F*_best_, the amplitude of which is the native amplitude |*F*_P_| multiplied by the figure of merit *m*, which is an estimate of the cosine of the phase error. Modern phasing programs now use maximum-likelihood methods that use advanced probability distributions that better model an experiment and thus obtain better estimates of parameters (Otwinowski, 1991 ▶; de La Fortelle & Bricogne, 1997 ▶; Pannu *et al.*, 2003 ▶; Pannu & Read, 2004 ▶). Such methods are employed in *MLPHARE* (Collaborative Computational Project, Number 4, 1994 ▶), *SHARP*, *BP*3 and *Phaser* (McCoy *et al.*, 2007 ▶).

**Figure 11:**
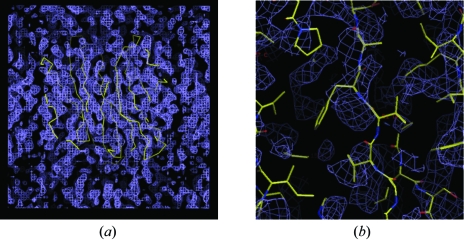
(*a*) An uninterpretable 2.6 Å SIR electron-density map with the final C^α^ trace of the structure superimposed. ρ(**x**) = (1/*V*)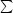 *m*|*F*_P_|exp(*i*α_best_)× exp(−2π*i* **h**·**x**). (*b*) A small section of the map with the final structure superimposed.

Fig. 11 ▶ shows the electron density of part of the unit cell of the sialidase from *Salmonella typhimurium* (Crennell *et al.*, 1993 ▶) phased using a single mercury derivative. Although the protein–solvent boundary is partly evident, the electron density remains uninterpretable.

**Figure 12:**
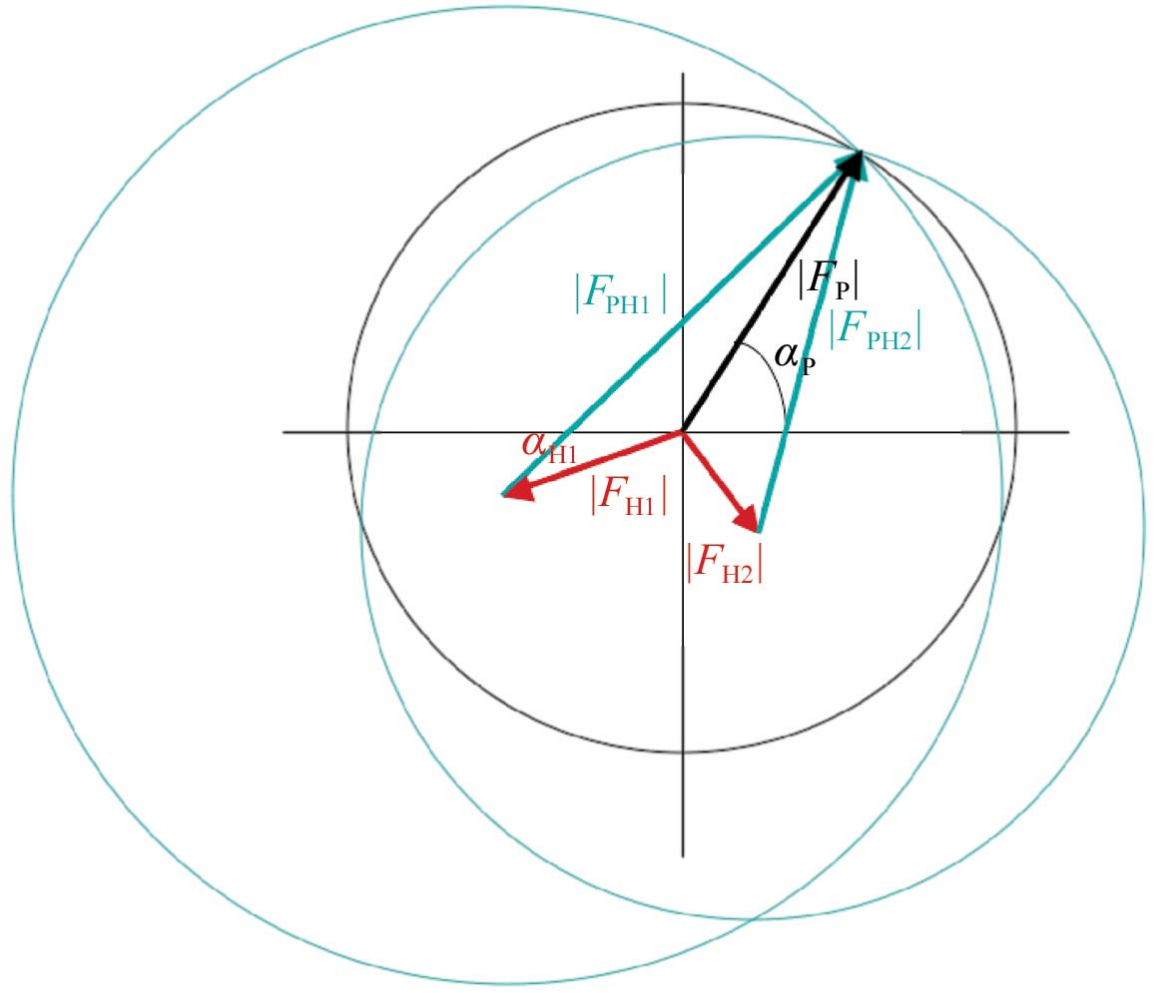
Harker diagram for MIR with two heavy-atom derivatives.

The use of more than one heavy-atom derivative in multiple isomorphous replacement (MIR) can break the phase ambiguity, as shown in Fig. 12 ▶ for a perfect case where the three circles overlap at one phase angle.

**Figure 13:**
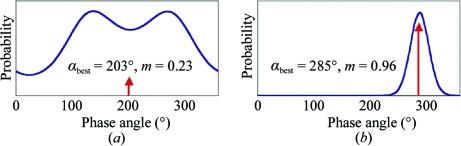
Phase probability for one reflection. (*a*) Single derivative in an SIR experiment. (*b*) Three derivatives. In an MIR experiment *P*(α_*P*_) ∝ Πexp(−∊_*i*_^2^/2*E*_*i*_^2^), where *i* is summed from 1 up to the number of derivatives.

The phase probability is obtained by multiplying the individual phase probabilities together, as shown in Fig. 13 ▶ for the same reflection as in Fig. 10 ▶, but this time three heavy-atom derivatives have resulted in a sharp unimodal distribution with a concomitantly high figure of merit.

## 4. Phase improvement

**Figure 14:**
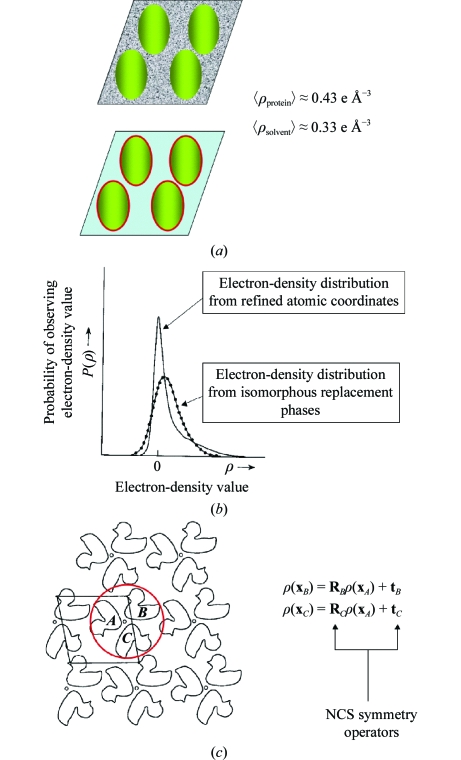
Density-modification techniques. (*a*) Solvent flattening uses automated methods to define the protein–solvent boundary and then modifies the solvent electron density to be a certain fixed value. (*b*) Histogram matching redefines the values of electron-density points in a map so that they conform to an expected distribution of electron-density values. (*c*) Noncrystallographic (NCS) symmetry averaging imposes identical electron-density values to points related by local symmetry, in this case a trimer of ducks that forms the asymmetric unit. The local NCS symmetry operators relating points in duck *A* to ducks *B* and *C* are shown.

It is rare that experimentally determined phases are sufficiently accurate to give a completely interpretable electron-density map. Experimental phases are usually the starting point for phase improvement using a variety of density-modification methods, which are also based on some prior knowledge of structure. Solvent flattening, solvent flipping, histogram matching and noncrystallographic averaging are the main techniques that are used to modify electron density and improve phases (Fig. 14 ▶). Solvent flattening is a powerful technique that removes negative electron density and sets the value of electron density in the solvent regions to a typical value of 0.33 e Å^−3^, in contrast to a typical protein electron density of 0.43 e Å^−3^. Automatic methods are used to define the protein–solvent boundary; they were initially developed by Wang (1985 ▶) and were extended into reciprocal space by Leslie (1988 ▶). A variation of this method that avoids the problem of bias introduced by iterative solvent flattening and phase combination is the so-called solvent-flipping method (Abrahams & Leslie, 1996 ▶). Histogram matching alters the values of electron-density points to concur with an expected distribution of electron-density values. Noncrystallographic symmetry averaging imposes equivalence on electron-density values when more than one copy of a molecule is present in the asymmetric unit. These methods were originally encoded into programs such as *DM* (Cowtan & Zhang, 1999 ▶), *RESOLVE* (Terwilliger, 2002 ▶) and *CNS* (Brünger *et al.*, 1998 ▶). Automatic interpretation of the electron-density map by tracing the main chain and side chains is another powerful method for improving phases. The program *ARP*/*wARP* is particularly useful and performs cycles of placing dummy atoms into electron-density maps followed by refinement, model building and update (Langer *et al.*, 2008 ▶). Similar methods are available in *RESOLVE*, particularly as part of the *PHENIX* suite of programs that cycle between phase improvement, model building and refinement (Adams *et al.*, 2002 ▶). For extensive automatic interpretation, including assignment of side chains, these methods generally require data to at least 2.7 Å resolution. However, other methods allow the identification of α-­helices and β-strands at lower resolution, such as Cowtan’s *Buccaneer* discussed elsewhere in this issue. In *SHELXE*, Sheldrick uses a characteristically novel approach to density modification (Sheldrick, 2008 ▶) and a more recent version of his program incorporates chain-tracing, again discussed elsewhere in this issue. Density-modification techniques will not turn a bad map into a good one, but they will certainly improve promising maps that show some interpretable features.

**Figure 15:**
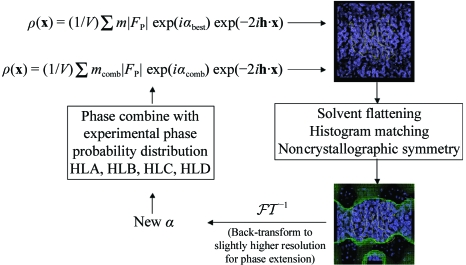
Phase improvement by density modification.

**Figure 16:**
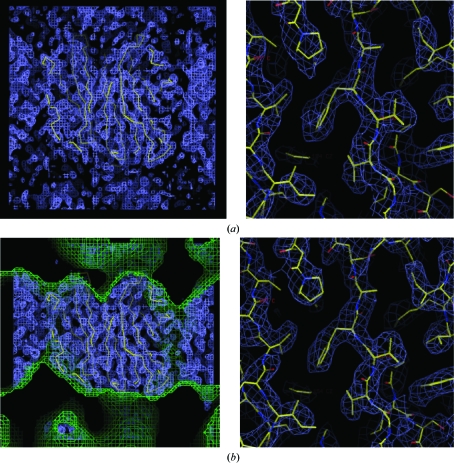
(*a*) 2.6 Å MIR electron density. (*b*) Electron density after solvent flattening and histogram matching in *DM*. The solvent envelope determined by *DM* is shown in green.

Density modification is a cyclic procedure, involving the back-transformation of the modified electron-density map to give modified phases, the recombination of these phases with the experimental phases (so as not to throw away experimental reality) and the calculation of a new map which is then modified and so the cycle continues to convergence. If native data have been collected to a higher resolution, such methods can also be used to provide phases beyond the resolution for which experimental phase information is available. In such cases, the modified map is back-transformed to a slightly higher resolution in each cycle to provide new phases for a subset of higher resolution reflections. The process is illustrated in Fig. 15 ▶. An example of the application of solvent flattening and histogram matching using *DM* is shown in Fig. 16 ▶ for the *S. typhimurium* sialidase phased on three derivatives.

## 5. Anomalous scattering

### 5.1. The anomalous scattering factor

**Figure 17:**
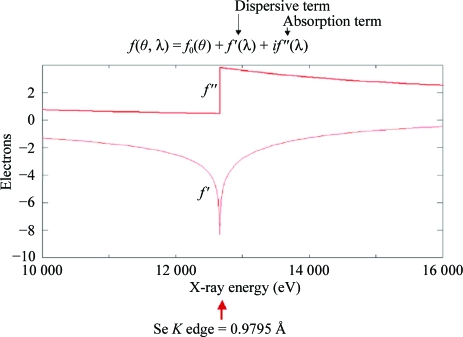
Variation in anomalous scattering signal *versus* incident X-ray energy in the vicinity of the *K* edge of selenium.

**Figure 18:**
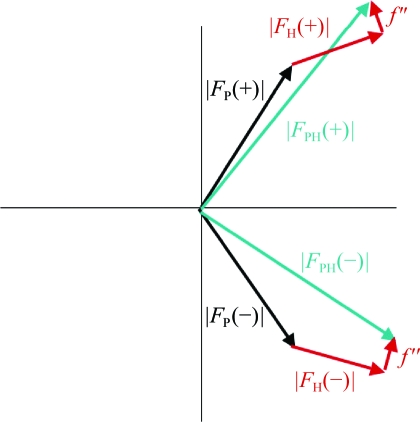
Breakdown of Friedel’s law when an anomalous scatterer is present. *f*(θ, λ) = *f*_0_(θ) + *f*′(λ) + *if*′′(λ). |*F*_*hkl*_| ≠ |*F*_−*h*−*k*−*l*_| or |*F*_PH(+)_| ≠ |*F*_PH(−)_|. Δ*F* ^±^ = |*F*_PH_(+)| − |F_PH_(−)| is the Bijvoet difference.

The atomic scattering factor contains three components: a normal scattering term *f*_0_ that is dependent on the Bragg angle and two terms *f*′ and *f*′′ that are not dependent on scattering angle but are dependent on wavelength. These latter two terms represent the anomalous scattering that occurs at the absorption edge when the X-ray photon energy is sufficient to promote an electron from an inner shell. The dispersive term *f*′ modifies the normal scattering factor, whereas the absorption term *f*′′ is 90° advanced in phase. Friedel’s law holds that |*F*_*hkl*_| = |*F*_−*h*−*k*−*l*_|; however, in the presence of an anomalous scatterer Friedel’s law breaks down, giving rise to anomalous differences that can be used to locate the anomalous scatterers. Fig. 17 ▶ shows the variation in anomalous scattering at the *K* edge of selenium and Fig. 18 ▶ shows the breakdown of Friedel’s law.

**Figure 19:**
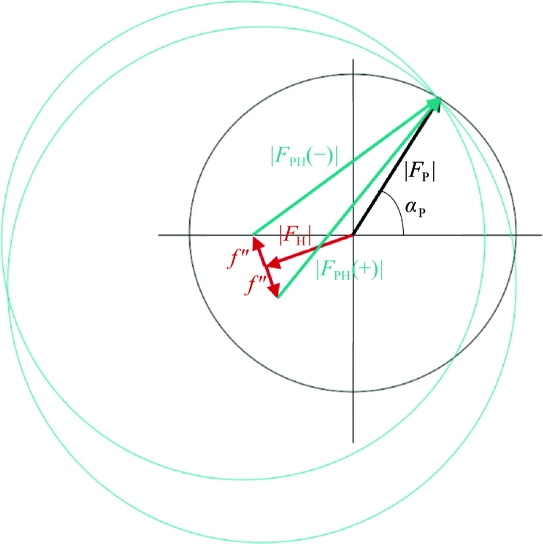
Harker construction for SIRAS.

The anomalous or Bijvoet difference can be used in the same way as the isomorphous difference in Patterson or direct methods to locate the anomalous scatterers. Phases for the native structure factors can then be derived in a similar way to the SIR or MIR case. Anomalous scattering can be used to break the phase ambiguity in a single isomorphous replacement experiment, leading to SIRAS (single isomorphous replacement with anomalous scattering). Note that because of the 90° phase advance of the *f*′′ term, anomalous scattering provides orthogonal phase information to the isomorphous term. In Fig. 19 ▶ there are two possible phase values symmetrically located about *f*′′ and two possible phase values symmetrically located about *F*_H_. MIRAS is the term used to describe multiple isomorphous heavy-atom replacement using anomalous scattering.

### 5.2. MAD

**Figure 20:**
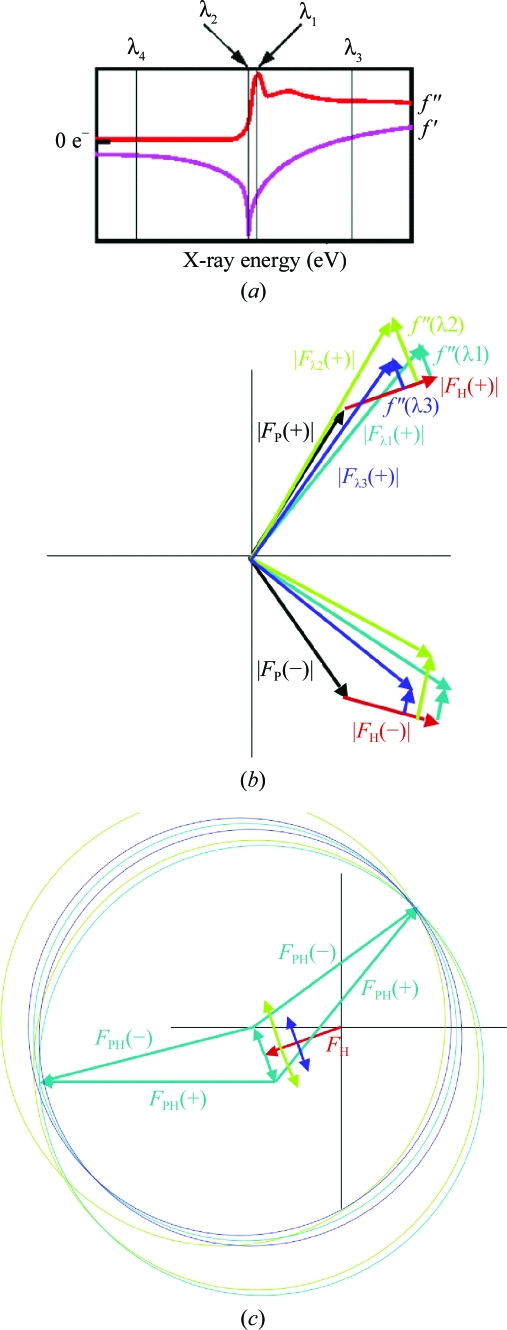
MAD phasing. (*a*) Typical absorption curve for an anomalous scatterer. (*b*) Phase diagram. |*F*_P_| is not measured, so one of the data sets is chosen as the ‘native’. (*c*) Harker construction.

Isomorphous replacement has several problems: non-isomorphism between crystals (unit-cell changes, reorientation of the protein, conformational changes, changes in salt and solvent ions), problems in locating all the heavy atoms, problems in refining heavy-atom positions, occupancies and thermal parameters and errors in intensity measurements. The use of the multiwavelength anomalous diffraction/dispersion (MAD) method can at least overcome the non-isomorphism problems if there is no significant radiation damage. Data are collected from a single crystal at several wavelengths, typically three, in order to maximize the absorption and dispersive effects. Usually, wavelengths are chosen at the absorption (*f*′′) peak (λ_1_), at the point of inflection on the absorption curve (λ_2_), where the dispersive term *f*′ (which is the derivative of the *f*′′ curve) has its minimum, and at a remote wavelength (λ_3_ and/or λ_4_) to maximize the dispersive difference to λ_2_. Fig. 20 ▶ shows a typical absorption curve for an anomalous scatterer, together with the phase and Harker diagrams.

**Figure 21:**
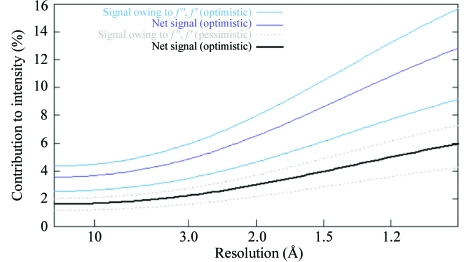
Estimation of signal size. The expected Bijvoet ratio is r.m.s.(Δ*F* ^±^)/r.m.s.(|*F*|) ≃ (*N*_A_/2*N*_T_)^1/2^(2*f*′′_A_/*Z*_eff_). The expected dispersive ratio is r.m.s.(Δ*F*_Δλ_)/r.m.s.(|*F*|) ≃ (*N*_A_/2*N*_T_)^1/2^[|*f*′_A_(λ_*i*_) - *f*′_A_(λ_*j*_)|]/*Z*_eff_, where *N*_A_ is the number of anomalous scatterers, *N*_T_ is the total number of atoms in the structure and *Z*_eff_ is the normal scattering power for all atoms (6.7 e^−^ at 2θ = 0).

The changes in structure-factor amplitudes arising from anomalous scattering are generally small and require accurate measurement of intensities. The actual shape of the absorption curve should be determined experimentally by a fluorescence scan on the crystal at the synchrotron, as the environment of the anomalous scatterers can affect the details of the absorption. There is a need for excellent optics to ensure accurate wavelength setting with a minimum of wavelength dispersion. Generally, all data are collected from a single cryocooled crystal with high multiplicity to increase the statistical significance of the measurements and data are collected with as high a completeness as possible. The signal size can be estimated using equations similar to those derived by Crick and Magdoff for isomorphous changes. Fig. 21 ▶ shows a predicted signal for the case of two Se atoms in 200 amino acids calculated using Ethan Merritt’s web-based calculator (http://www.bmsc.washington.edu/scatter/AS_index.html). Note that the signal increases with resolution.

### 5.3. SAD

**Figure 22:**
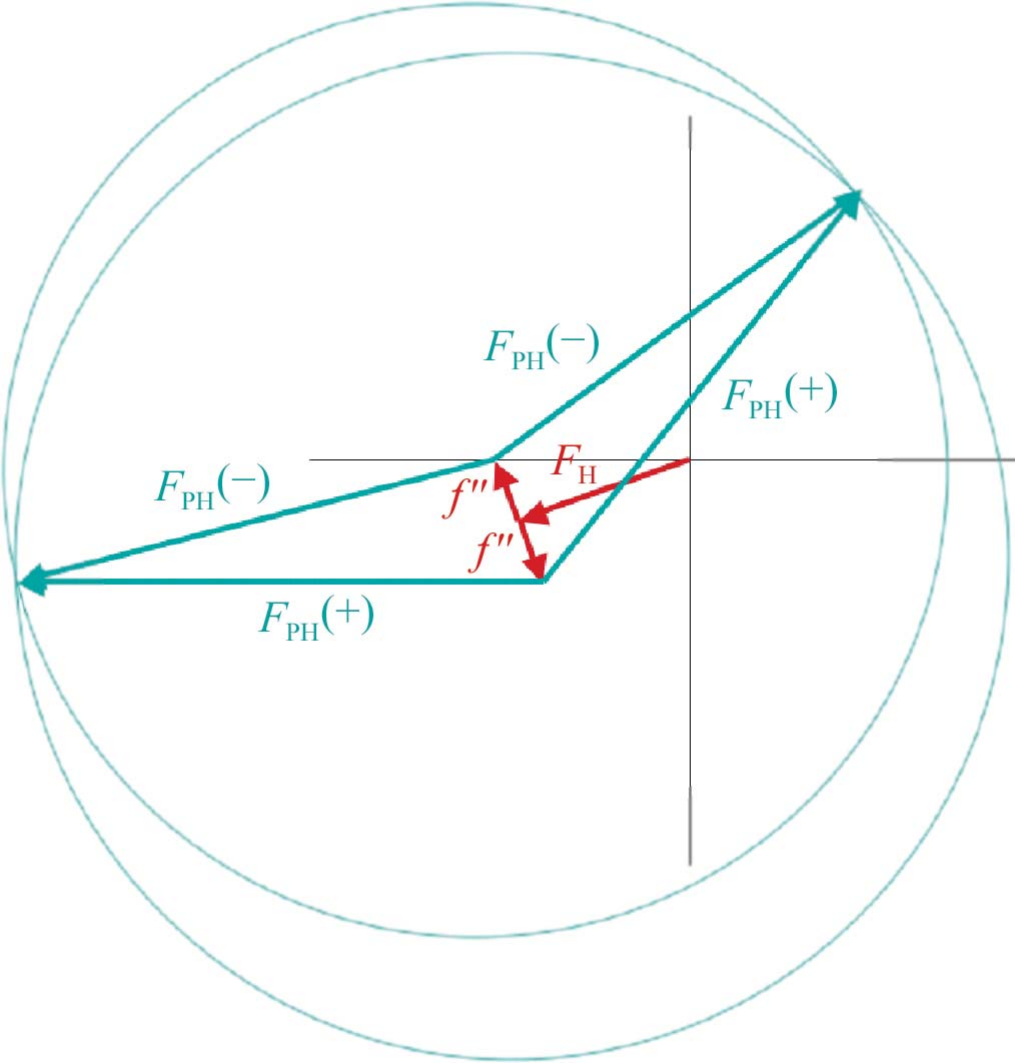
Harker construction for SAD.

Increasing numbers of protein structures are now being phased using only a single set of diffraction data by the single-wavelength anomalous dispersion/diffraction (SAD) method (Wang, 1985 ▶). The first demonstration of this was for the 46-­residue protein crambin, which was phased with six intrinsic sulfurs using in-house data collected at the Cu *K*α wavelength (Hendrickson & Teeter, 1981 ▶). Subsequently, it was demonstrated for the 129-residue hen egg-white lysozyme (Dauter *et al.*, 1999 ▶) and the method has now become routine (Dauter *et al.*, 2002 ▶; Dodson, 2003 ▶). The SAD experiment only provides measurements of the anomalous, or Bijvoet, differences Δ*F* ^±^ = |*F*_PH_(+)| − | *F*_PH_(−)|. These are then used as estimates of the heavy-atom contribution to the scattering and enable direct or Patterson methods to be used to derive the positions of the heavy-atom substructure. The Harker con­struction for a single reflection from a hypothetical SAD experiment (Fig. 22 ▶) shows that once the heavy-atom sub­structure is known the calculated amplitude and phase of this contribution can be drawn (*F*_H_). However, an ambiguity remains in the phase of the protein structure factor, with values symmetrically located around the absorption contribution (*f*′) to the anomalous scattering. This phase ambiguity has to be broken through density-modification procedures, which have become much more powerful in recent years. In its purest form, SAD can simply utilize the intrinsic anomalous scatterers present in the macromolecule, such as the S atoms of cysteine and methionine or bound ions. The challenge is in maximizing and measuring the very small signal, since the Bijvoet ratio can be as low as 1% when the typical merging *R* factor is several times this value. The trick lies in making multiple measurements of reflections at an appropriate wavelength in order to achieve a high multiplicity that will give statistically accurate measurements of the anomalous difference. The data should also be as complete as possible.

There has been much discussion of data-collection strategies, scaling protocols and the best wavelength at which to collect data. A fascinating and comprehensive study from a group at EMBL Hamburg showed that a wavelength of ∼2 Å gave the maximum anomalous signal for a range of proteins containing anomalous scatterers such as S, P, Ca, Xe, Cl or Zn (Mueller-Dieckmann *et al.*, 2007 ▶). The availability of Cr *K*α radiation, which has a wavelength of 2.29 Å, is leading to the use of chromium anodes for in-house phasing of macromolecules based on S (Yang *et al.*, 2003 ▶; Watanabe *et al.*, 2005 ▶) or Se atoms (Xu *et al.*, 2005 ▶).

Two examples are now given that show the power of the SAD method. The first involves phasing based on S atoms (S-­SAD) and the second is based on phasing from a single Hg atom (Hg-SAD). The data sets and tutorial guides can be found at http://www.st-andrews.ac.uk/~glt2/CCP4 for those who wish to experiment with the data handling and structure solution.

### 5.4. S-SAD example

**Figure 23:**
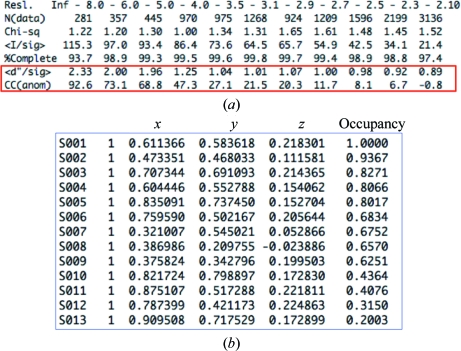
(*a*) Statistics from *SHELXC* showing the anomalous signal for the S-SAD example. (*b*) Heavy-atom sites determined by *SHELXD*.

This example uses highly accurate S-SAD data collected to a resolution of 2.1 Å on beamline BM14 of the ESRF at a wavelength of 1.722 Å. Two orientations of the crystal were used to collect 760° of data with 30-fold multiplicity. The merging *R* factor of the data was 0.067 overall and was 0.252 in the highest resolution shell. The protein consists of 238 residues (27.3 kDa) and contains nine methionines and no cysteines, giving an estimated signal of 1% for the Bijvoet ratio (Δ*F* ^±^/*F*; http://www.ruppweb.org/new_comp/anomalous_scattering.htm). If the data had been collected in-house using Cu *K*α radiation the signal would have been ∼0.8%, whereas if data were collected at the *K* edge of sulfur (∼5 Å wavelength) the signal would be 6%. There are many practical reasons why collecting data at such a long wavelength is not viable, for example air absorption and the spreading out of the diffraction pattern. A high-resolution data set was also collected at the ESRF to a resolution of 1.45 Å at a wavelength of 0.9762 Å. The crystals belonged to space group *P*2_1_2_1_2_1_, with one molecule in the asymmetric unit and an estimated solvent content of 40%. *SHELXC* was used to read the scaled unmerged intensity data processed using *HKL*-2000 (Otwinowski & Minor, 1997 ▶) and to prepare a list of heavy-atom structure-factor estimates derived from the anomalous differences. The statistics of the S-SAD data are shown in Fig. 23 ▶ and suggest that the anomalous signal [〈*d*′′/sig〉 or 〈(Δ*F* ^±^)/σ(Δ*F* ^±^)〉] is detectable to about 2.7 Å. *SHELXD* (Sheldrick, 2008 ▶) was then used with data to 2.7 Å resolution to find the substructure of anomalous scatterers. *SHELXE* (Sheldrick, 2008 ▶) was used to calculate the centroid phases from the Harker construction and to perform density modification to break the phase ambiguity. Note that both hands of the heavy atoms need to be tried, as an arbitrary choice of hand is made in the determination of the heavy-atom positions. In *SHELXE* this simply requires running the pro­gram again with an extra switch to reverse the hand. *SHELXD* appears to have found all nine sulfur sites and four additional sites that may be occupied by solvent ions (Fig. 23 ▶).

**Figure 24:**
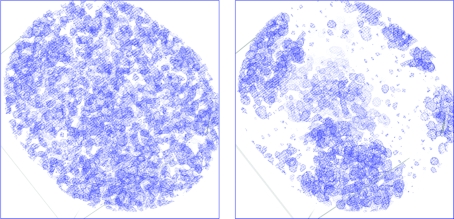
2.1 Å electron-density map for the S-SAD example before and after density modification using *SHELXE*.

**Figure 25:**
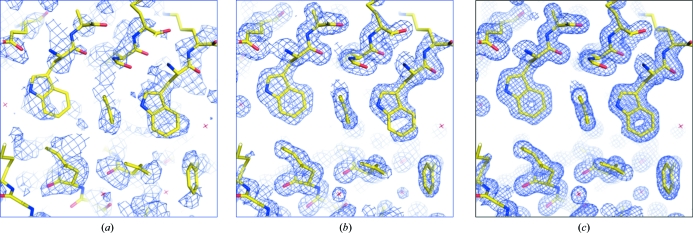
Improving phases for the S-SAD problem. (*a*) 2.1 Å resolution density-modified map. (*b*) 1.45 Å resolution phase-extended map. (*c*) ‘1.0 Å resolution’ free-lunch map.

**Figure 26:**
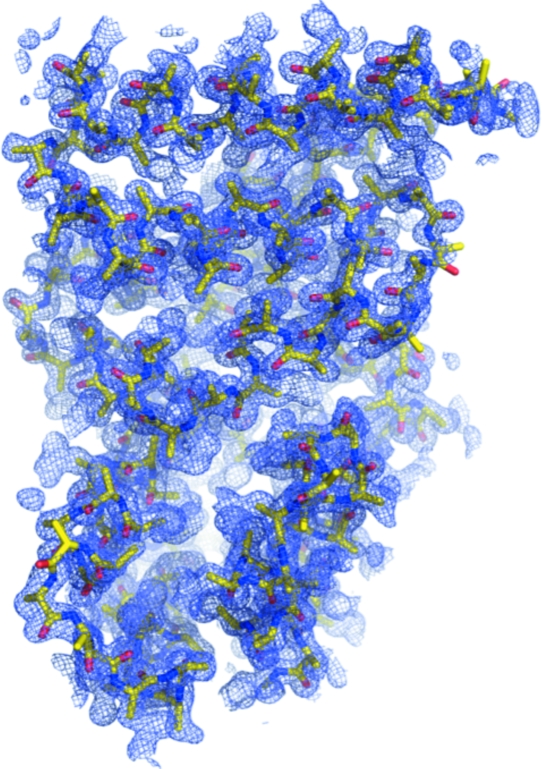
Autotraced polyalanine model produced by *SHELXE* superimposed on the density-modified electron-density map at 1.45 Å resolution.

The electron-density maps at 2.1 Å calculated using the phases derived from these heavy atoms before and after density modification are shown in Fig. 24 ▶ and the latter clearly shows the protein–solvent boundary after density modification. Incorporation of the 1.45 Å data into *SHELXE* allowed phase extension to provide a highly interpretable map (Fig. 25 ▶ *b*). If data are available to at least 2.0 Å resolution then the ‘free-lunch’ algorithm in *SHELXE* can be invoked (Usón *et al.*, 2007 ▶). In this case, as data were available to 1.45 Å, phases were calculated to 1.0 Å using the free-lunch algorithm, producing a remarkable map from which the sequence of the protein could be easily read (Fig. 25 ▶ *c*). Note that this is not a real 1.0 Å map, as the extended data have been generated and not experimentally derived, but the free-lunch algorithm can be a powerful tool to improve the phases of experimentally measured data. Finally, the latest version of *SHELX* incorporates an autotracing algorithm that attempts to create a polyalanine model (shown in Fig. 26) ▶, the main use of which is to further improve the phases. *SHELXE* built 160 residues into the map, far less than the 238 residues expected; however, the first 60 residues of this protein are disordered and are not visible in the electron density. In this S-SAD example, the final phases from *SHELXE* were used to automatically build a model fitted to the sequence using *ARP*/*wARP* (Cohen *et al.*, 2008 ▶).

### 5.5. Hg-SAD example

**Figure 27:**
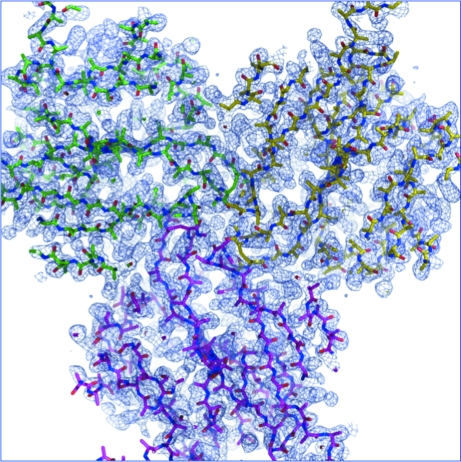
A *SHELXE*-derived 2.1 Å resolution electron-density map phased from a Hg-SAD data set with superimposed polyalanine trace produced by *SHELXE*. The view is down the crystallographic threefold axis.

The second example involves data that were collected in-house from a Hg-derivatized protein of 440 residues using Cu *K*α radiation. The structure was actually solved using SIRAS (Xu *et al.*, 2009 ▶), but it is interesting to note that the structure could have been solved using just the anomalous scattering information in the Hg-derivative data set. This example shows that it is worth looking at the phasing from a single-derivative data set in instances where the derivative is non-isomorphous with the native. The Hg derivative diffracted to 2.1 Å resolution and a data set was collected with only fourfold multiplicity. The cubic crystals belonged to space group *P*2_1_3, with unit-cell parameter *a* = 125.3 Å, and had a monomer in the asymmetric unit and a solvent content of 64%. The protein contained one Hg atom per monomer, giving an estimated Bijvoet ratio of 2.7% for Cu *K*α (1.54 Å), only slightly less than the signal of 3.6% that would be obtained at the Hg *L*_III_ edge (1.009 Å). *SHELXC* showed that the anomalous signal was present to ∼3.2 Å; therefore, data limited to this resolution were input into *SHELXD*, which readily found the single Hg site. *SHELXE* was used to determine the phases to 2.1 Å resolution and density modification with autotracing in *SHELXE* produced a polyalanine model that consisted of 389 of the 432 ordered residues of the final model (Fig. 27 ▶).

## 6. Cross-crystal averaging

**Figure 28:**
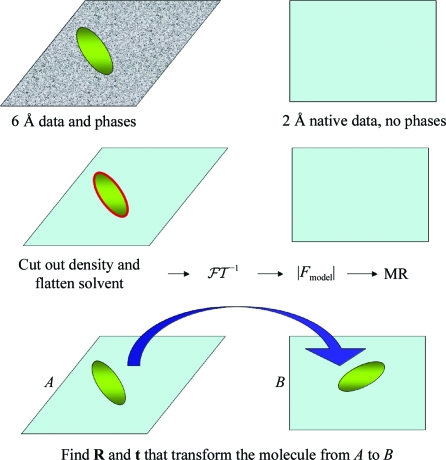
Cross-crystal averaging. Two crystal forms of the same protein for which phase information to low resolution is known for one form (left) and high-resolution data exist but no phase information is known for another form (right).

Protein crystallography is not a black-box technique for every protein; there are still challenges to be met in cases where MAD or SAD techniques cannot be used to derive a high-resolution map. On occasion two or more crystal forms of a protein are available, where low-resolution phases may be available for one crystal form but high-resolution data are available for another crystal form. Cross-crystal averaging involves mapping the electron density from the one unit cell into the other. Phases can then be derived for the new crystal form and through averaging of density between crystal forms and possibly phase extension as part of a density-modification procedure one can bootstrap the phases to high resolution. The procedure is outlined in Fig. 28 ▶.

**Figure 29:**
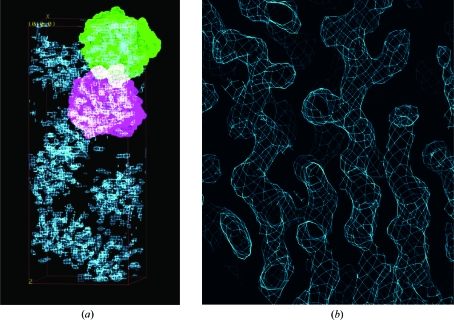
Cross-crystal averaging of hemagglutinin–neuraminidase (HN). Left, the unit cell showing the 6.0 Å resolution MIR map derived from eight heavy-atom derivatives contoured at 2.0σ, revealing two blobs corresponding to the two molecules in the asymmetric unit. Right, a section of the 2.0 Å resolution map after phase extension and cross-crystal averaging over four non-isomorphous data sets.

One example of the power of cross-crystal averaging is that of Newcastle disease virus haemagglutinin–neuraminidase (HN), the structure solution of which was plagued with non-isomorphism problems (Crennell *et al.*, 2000 ▶). Native crystals from the same crystallization drop could have significantly different unit-cell dimensions. The protein was derived from virus grown in embryonated chicken eggs, so SeMet methods were out of the question. Most heavy-atom derivatives were non-isomorphous with the native crystals and with one another. A platinum derivative was found that gave a clear peak in an anomalous Patterson, which led to an attempt at MAD phasing, but the signal was just too small. The *P*2_1_2_1_2_1_ unit cell had dimensions that varied as follows: *a* = 70.7–74.5, *b* = 71.8–87.0, *c* = 194.6–205.4 Å. In the end, cross-crystal averaging was used to bootstrap from a poor uninterpretable 6.0 Å resolution MIR map out to a clearly interpretable 2.0 Å resolution map (Fig. 29 ▶). Four data sets were chosen for cross-crystal averaging in *DMMULTI* and were chosen on the criteria that they were (i) as non-isomorphous as possible to one another and (ii) at as high a resolution as possible. These were a pH 7 room-temperature data set to 2.8 Å resolution (*a* = 73.3, *b* = 78.0, *c* = 202.6 Å), for which MIR phases were available to 6.0 Å, a pH 6 room-temperature data set to 3.0 Å resolution (*a* = 72.0, *b* = 83.9, *c* = 201.6 Å), a pH 4.6 cryocooled data set to 2.5 Å resolution (*a* = 71.7, *b* = 77.9, *c* = 198.2 Å) and a pH 4.6 cryocooled data set to 2.0 Å resolution (*a* = 72.3, *b* = 78.1, *c* = 199.4 Å). The power of the method lies in the fact that the different unit cells are sampling the molecular transform at different places. Like most things the idea is not new and was indeed used by Bragg and Perutz in the early days of haemoglobin (Bragg & Perutz, 1952 ▶), when they altered the unit cell of the crystals by controlled dehydration in order to sample the one-dimensional transform of the molecules in the unit cell. This paper is worth a read, if only for the wonderful inclusion of random test data in the form of train times between London and Cambridge!

## 7. Conclusion

The phase problem is fundamental and will never go away; however, its solution is now fairly routine thanks to MR, MAD and SAD. The wider availability of synchrotron sources, improvements in detector technologies, cryocrystallography and the development of more sophisticated software packages have contributed to the routine use of MAD, and increasingly SAD, to phase novel macromolecular structures within minutes of collecting the diffraction data. SAD is an unfortunate acronym for a method that can bring immense joy to the structural biologist!

## Figures and Tables

**Table 1:** Methods used in structural solution

Method	Prior knowledge
Direct methods	ρ ≥ 0, discrete atoms
Molecular replacement	Structurally similar model
Isomorphous replacement	Heavy-atom substructure
Anomalous scattering	Anomalous-atom substructure
	
Density modification	Solvent flattening
(phase improvement)	Histogram matching
	Noncrystallographic symmetry averaging
	Automatic partial structure detection
	Phase extension
